# Predictors of secondary HIV transmission risk in a cohort of adolescents living with HIV in South Africa

**DOI:** 10.1097/QAD.0000000000003044

**Published:** 2021-08-09

**Authors:** Elona Toska, Siyanai Zhou, Christina A. Laurenzi, Roxanna Haghighat, Wylene Saal, Laurie Gulaid, Lucie Cluver

**Affiliations:** a Centre for Social Science Research; b Department of Sociology, University of Cape Town, Cape Town, South Africa; c Department of Social Policy and Intervention, University of Oxford, Oxford, UK; d Institute for Life Course Health Research, Department of Global Health, Stellenbosch University; e UNICEF Eastern and Southern Africa Regional Office; f Department of Psychiatry and Mental Health, University of Cape Town, Cape Town, South Africa.

**Keywords:** adolescents, HIV, screening, secondary transmission, South Africa, young people

## Abstract

Preventing secondary HIV transmission from adolescents and young people living with HIV (AYPLHIV) to their partners and children is critical to interrupting the HIV infection cycle in sub-Saharan Africa. We investigated predictors of secondary HIV transmission risk (past-year sexual risk combined with past-year viremia) among AYPLHIV in South Africa.

**Design:**

A prospective cohort of AYLPHIV in South Africa recruited *n* = 1046 participants in 2014–2015, 93.6% of whom were followed up in 2016–2017 (1.5% mortality). Questionnaires used validated scales where available and biomarkers were extracted from *n* = 67 health facilities.

**Methods:**

Multivariate logistic regressions tested baseline factors associated with secondary HIV transmission risk, controlling for covariates, with marginal effect modelling combinations.

**Results:**

About 14.2% of AYPLHIV reported high secondary HIV transmission risk. High-risk AYPLHIV were more likely to be sexually infected [adjusted odds ratio (aOR) 2.79, 95% confidence interval (95% CI) 1.66–4.68, *P* < 0.001], and report hunger (aOR 1.93, 95% CI 1.18–3.14, *P* = 0.008) and substance use (aOR 2.19, 95% CI 1.19–4.02, *P* = 0.012). They were more likely to be in power-inequitable relationships (aOR 1.77, 95% CI 1.08–2.92, *P* = 0.025) and be parents (aOR 4.30, 95% CI 2.16–8.57, *P* < 0.001). Adolescents reporting none of these factors had a 4% probability of secondary transmission risk, rising to 89% probability with all five identified factors. Older age and early sexual debut were also strongly associated with a higher risk of secondary HIV transmission.

**Conclusion:**

It is essential to identify and support AYPLHIV at a high risk of secondary transmission. Screening for factors such as mode of infection and parenthood during routine healthcare visits could help identify and provide resources to the most at-risk adolescents.

## Introduction

Adolescents and young people living with HIV (AYPLHIV) are central to our HIV prevention agenda. Adolescents have lower rates of antiretroviral treatment (ART) adherence and poorer treatment outcomes than both children and adults [1–3]. Moreover, adolescence is a life-stage characterized by exploring relationships and testing boundaries. As AYPLHIV enter adulthood, explore sexual and romantic relationships, and initiate childbearing [4], they also face three interrelated risks: the risk of reinfection with other strains of the virus [5], the risk of their sexual partners becoming infected with HIV (secondary transmission) [6] and increased risks that their children will become infected with HIV [7–9].

Early data from an HIV prevention trial in Rakai, Uganda, in the late 1990s found that transmission risk rates were highest among 15 to 19-year-old adolescents living with HIV compared with older participants [10]. However, no quantitative studies to date have focused on examining factors associated with secondary transmission among AYPLHIV in sub-Saharan Africa. There is some evidence on proxy measures of secondary transmission risk individually: sexual risk or nonadherence/viral suppression. A growing body of research has documented factors associated with ART nonadherence and lack of viral suppression in this age group [11–13], including substance use [13,14] and complex romantic and sexual relationships [15,16]. There is mixed evidence surrounding potential predictors of sexual risk-taking among AYPLHIV, with no longitudinal data from sub-Saharan Africa. Several cross-sectional Africa-based studies have identified factors that are associated with sexual risk-taking, including older age, rural residence [17,18], parental monitoring, vertical infection [17], substance use [19,20] and power-inequitable sexual relationships in adolescence [21–23]. But transmission risk is substantially increased by the combination of sexual risk-taking and detectable viral load. No known studies in sub-Saharan Africa have reported on composite risk measures, accounting for both high-risk sex and viral load levels [17,24,25].

Identifying AYPLHIV at risk of secondary transmission is essential in order to interrupt the HIV transmission cycle. AYPLHIV in resource-constrained settings have limited access to timely viral load data [26] and rarely receive integrated HIV and sexual and reproductive health (SRH) services [27]. Therefore, timely biomedical data on whether adolescents are at risk of secondary HIV transmission may not be available, particularly in resource-constrained settings. Siloed service provision may also miss opportunities to identify and link to services those AYPLHIV who are most at risk. As such, tools to identify and support AYPLHIV who may be at risk of secondary transmission, especially in resource-constrained settings, are urgently needed. To address this gap, we examined factors associated with an increased risk of secondary transmission among AYPLHIV in South Africa, sing data from a two-wave community-traced study, testing hypothesized factors that could be feasibly screened for during routine healthcare visits.

## Materials and methods

### Study design and participants

This study is a prospective cohort of AYPLHIV, Mzantsi Wakho, conducted in the Eastern Cape Province in South Africa. The study catchment area is a mixed rural-urban health sub-district with an estimated HIV prevalence of 13.6% [28]. Participants (*n* = 1046), including all ART-initiated 10 to 19-year-olds from 52 public health facilities, were recruited at baseline in 2014–2015 (90% of all eligible participants). At follow-up, *n* = 979 were reinterviewed (93.6% retention, 1.5% mortality, 1.4% refusals, 3.1% untraceable). To reduce recruitment bias, this study included adolescents not engaged in medical care by tracing participants in their communities (>180 villages, neighbourhoods and settlements). To minimize HIV-related stigma resulting from study participation, an additional *n* = 467 cohabiting adolescents were interviewed with non-HIV specific questionnaires (excluded from this analysis).

Informed written adolescent consent was obtained alongside caregiver consent for minors prior to study participation for both rounds of data collection. Experienced research assistants read consent forms carefully in the local language (Xhosa) or English to ensure full comprehension, even in cases of low literacy. Questionnaires were administered in English or Xhosa, based on participant preference, by highly trained researchers with experience working with vulnerable children and adolescents. In parallel, clinic-based researchers extracted participants’ clinical records from 67 health facilities (primary clinics, community health centres and hospitals) in 2014–2017, following participant and caregiver consent. Participants’ records across multiple facilities were individually linked using unique study identifiers.

The study was developed in collaboration with South African National Departments of Health, Basic Education, Social Development; the South African National AIDS Council; UNICEF; other implementing partners including Paediatric-Adolescent Treatment Africa; and consultations with AYPLHIV. Ethical approvals were obtained from University of Oxford (SSD/CUREC2/12-21), University of Cape Town (UCT/CSSR/2019/01), the provincial Departments of Health and Basic Education, and participating health facility ethics committees.

### Measures

The main outcome in this study was *secondary transmission risk*. At both baseline and follow-up, risk was defined as the proportion of participants with past-year viremia, and reported past-year sexual risk. *Past-year viremia* was computed using *detectable viral load* defined as a viral load more than 1500 copies/ml at last measurement informed by the sexual transmission rates documented in Rakai, Uganda [10] (for participants with a viral load record) or *past-week ART nonadherence* (for participants with missing viral load data). These two measures were combined given poor viral load coverage in the sample [26] and strong associations between high viral load, and self-reported defaulting and nonadherence, in sub-sample analyses [17] (see Tables 1 and 2, Supplementary Digital Content 1). *Past-year sexual risk* was computed on the basis of adolescent self-report of one or more of the following: unprotected sex at last intercourse, ever had transactional sex, multiple sexual partners in the last year, last sexual partner was 5 or more years older, and ever been pregnant or made someone pregnant, all of which were adapted from a nationally representative adolescent study [29]. At each timepoint, a composite transmission risk measure of both viremia and sexual risk was computed. Each participant was allocated to one of two groups: AYPLHIV calculated as having no transmission risk at both baseline and follow-up (*low transmission risk*) were compared with all other adolescents (*high transmission risk*).

**Table 1:** Sexual risk factors and viremia at baseline and follow-up.

	Full baseline sample (*N* = 1046)	Follow-up sample (*n* = 979)
Variables contributing to outcome measure	*N* (%)	*N* (%)
Sexual risk factors		
Transactional sex	68 (6.5%)	73 (7.5%)
Sex with older partner	26 (2.5%)	60 (6.1%)
Unprotected sex	65 (6.2%)	91 (9.3%)
Multiple sexual partners	91 (8.7%)	134 (13.7%)
Pregnancy	75 (7.2%)	98 (10.0%)
*Any sexual risk (combined)*	*158 (15.1%)*	*271 (27.7%)*
Treatment-related outcomes		
Detectable viral load (>200 copies/ml)^b^	167 (29.0%)	146 (28.0%)
Detectable viral load (>1500 copies/ml)^b^	124 (23.0%)	98 (18.8%)
Past-week nonadherence	365 (34.9%)	348 (35.6%)
*Viraemia* ^a^	*318 (30.4%)*	*288 (29.4%)*
Secondary HIV transmission risk^c^		
Sexual risk and viremia	70 (6.7%)	110 (11.2%)
Low risk (no risk at baseline nor follow-up)	840 (85.8%)	
High-risk group	139 (14.2%)	

a Combination of past-week nonadherence for those without VL record and detectable viral load (>1500 copies/ml) for those with VL record.

b Viral load data were available for 540 at baseline and 522 at follow-up. Frequencies of detectable viral load computed on available data.

c Secondary HIV transmission risk^c^ computed as participants reporting both sexual risk and viremia at each time point.

**Table 2:** Descriptive statistics for adolescents living with HIV, grouped by low and high risk of onwards transmission (*n* = 979).

	Transmission risk groups (*N* = 979)			
	Low risk (*N* = 840)	High risk (*N* = 139)	Total (*N* = 979)	
Baseline characteristics	*N* (%)	*N* (%)	*N* (%)	*P*
Sociodemographics				
Age binary (15+ years)	250 (29.8%)	112 (80.6%)	362 (37.0%)	<0.001
Sex (female)	441 (52.5%)	98 (70.5%)	539 (55.1%)	<0.001
Rural residence	224 (26.7%)	37 (26.8%)	261 (26.7%)	0.98
Poverty	659 (78.5%)	124 (89.2%)	783 (80.0%)	0.003
Double orphanhood	126 (15.0%)	29 (20.9%)	155 (15.8%)	0.079
School absenteeism (≥1 week)	154 (18.3%)	57 (41.0%)	211 (21.6%)	<0.001
Individual-level factors				
Negative peer norms (mean, SD)	0.62 (1.37)	2.30 (2.08)	2.92 (.3%)	<0.001
Internalized stigma	624 (74.3%)	108 (77.7%)	732 (74.8%)	0.39
Suicidality	26 (3.1%)	14 (10.1%)	40 (4.1%)	<0.001
Substance/drug use	42 (5.0%)	42 (30.2%)	84 (8.6%)	<0.001
Family factors				
Household food insecurity	166 (19.8%)	56 (40.3%)	222 (22.7%)	<0.001
Positive caregiving	433 (51.5%)	62 (44.6%)	495 (50.6%)	0.13
Good caregiver monitoring	374 (44.5%)	31 (22.3%)	405 (41.4%)	<0.001
Good adolescent-caregiver communication	219 (26.1%)	43 (30.9%)	262 (26.8%)	0.23
HIV-related factors				
Time on treatment (3+ years)	601 (72.8%)	62 (48.8%)	663 (67.7%)	<0.001
Knows HIV status	540 (65.2%)	122 (90.4%)	662 (67.6%)	<0.001
Treatment buddy support	628 (74.8%)	74 (53.2%)	702 (71.7%)	<0.001
Sexually infected	117 (14.0%)	86 (64.2%)	203 (20.7%)	<0.001
Relationship factors				
Early sexual debut	68 (8.1%)	61 (43.9%)	129 (13.2%)	<0.001
Parenthood	18 (2.1%)	48 (34.5%)	66 (6.7%)	<0.001
Power-inequitable relationships	126 (15.0%)	70 (50.4%)	196 (20.0%)	<0.001

Baseline measures of the following factors were included in the analyses:

(1) Sociodemographic factors included adolescent *age* [coded as younger (ages 10–14) and older (ages 15–19)]; *sex* (male/female); *urban/rural residence,* using census definitions [30]; *housing type* (informal/formal); *household poverty,* measured as missing one of seven basic necessities for children and adolescents validated in a nationally representative survey [31]; and *double orphanhood (both maternal and paternal)*, recorded using items developed from UNICEF. *Past-term school absenteeism* measured the number of days the adolescent missed school in the last full school term.
(2) Individual-level factors included *negative peer norms*, measured through a series of items assessing peer support for unsafe sex and adolescent pregnancy [32] and *mental health challenges,* including internalized stigma and suicidality. Internalized stigma was measured as a score more than 1 using the internalized stigma sub-scale of the ALHIV-SS, a locally adapted and validated stigma scale [33]. Suicidality was recorded as whether the adolescent had thought of a way, or tried, to kill him/herself [34]. *Substance use* was measured using an item adapted from WHO's AUDIT scale reporting if the adolescent's substance use interfered with walking, talking or memory, combined with an item derived with our adolescent advisory group (’I drink alcohol to have fun, without my caregivers knowing or approving’), validated with similar populations in South Africa [35].
(3) Family-level factors included *positive caregiving* using a six-item sub-scale from the Alabama Parenting Questionnaire [36], including warmth and praise from a primary caregiver; *good caregiver monitoring (supervision)* using a scale of 10 items from the relevant subscale of the Alabama Parenting Questionnaire, such as setting rules for times to come home; and *good adolescent-caregiver communication* using a scale of five items including openness and talking to the caregiver without fear. *Food insecurity* (*hunger)* was defined as a binary indicator of having enough food at home in the past week, not engaging in transactional sex for food, and not missing ART because of insufficient food.
(4) HIV-specific factors included *time on treatment* coded as more than 1 year on treatment, *knowledge of HIV-positive status* based on items developed through participatory research with AYPLHIV [37], *having a treatment buddy* and *mode of infection.* Adolescents who started treatment before age 10 were designated as vertically infected, similar to existing sub-Saharan African paediatric HIV cohorts [38], validated through an algorithm reported elsewhere [39].

Relationship factors included *early sexual debut* (<age 16); *having one or more child*; and *power-inequitable relationships*. Power-inequitable relationships were computed if an adolescent was in a relationship, and if they described self-reported inability to negotiate well tolerated sex in a relationship, nondisclosure of HIV status to sexual partner or inability to take ART while in the relationship (measured through an item adapted from the Adolescent Trials Network Group) [40].

### Analysis

We used multivariate logistic regression analysis to examine *secondary transmission risk* among AYPLHIV and associated factors. Analyses, conducted in Stata 15.1 (StataCorp LLC., College Station, Texas, USA; 2017), consisted of four steps. First, individual past-year sexual risk factors and viremia were computed for all participants, providing overall rates of secondary transmission risk. Second, frequencies of all key variables were computed comparing between adolescents at low and high HIV-transmission risk using chi-square tests for binary variables and one-way ANOVA for continuous variables. Third, stepwise multivariate regression models were run with *high secondary transmission risk* as an outcome, including all baseline individual, HIV and relationship-level predictors, controlling for baseline sociodemographic characteristics. In the first model, all potential predictors and covariates were included. In the second model, only factors significant at the 10% level (*P* < 0.1) were retained. In the third model, only factors significant at (*P* < 0.05) were entered, to maximize analysis power while taking potential covariates into account. Multicollinearity was assessed for all potential predictors and covariates using the Spearman's correlation and variance inflation factors (using *vif* command). A cut-off value of a strong correlation (±0.8) or VIF at least 10 was used in this analysis (see Table 4, Supplementary Digital Content 1) [41,42]. Given high levels of missing viral load data in participants’ clinical records, sensitivity analyses were conducted by repeating the primary analysis using different definitions of detectable viral load thresholds for viremia: more than 200, more than 400 and more than 1000 copies/ml (results not shown). Fourth, marginal effect modelling was used to compute probabilities of reporting the outcome under different combinations of factors significant in the final multivariate regression. We modelled the effect of factors that could be screened for during routine healthcare visits, to inform potential tools that can be tested and used by healthcare providers or peer supporters that were significant in the final regression model in the prior step.

## Results

Successfully followed-up participants were more likely to be younger, live in rural areas and have experienced orphanhood, compared with their lost-to-follow-up counterparts, as reported elsewhere [17]. Only participants included in both waves were included in analyses, so data reported in this manuscript focuses primarily on the sample included at both time points of *n* = 979.

### High secondary transmission risk

**Fig. 1:**
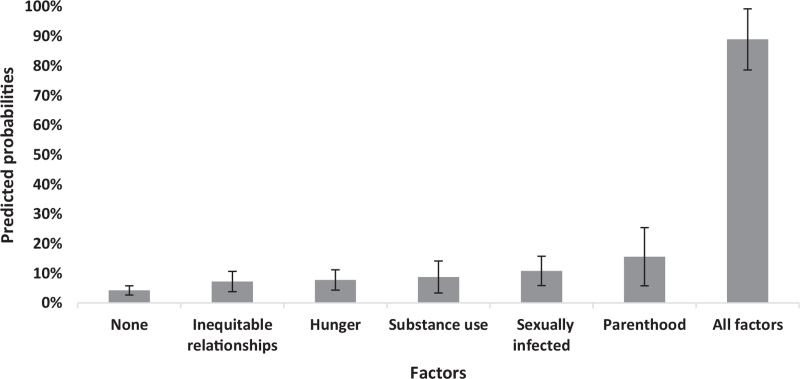
Probability of high secondary HIV transmission risk (controlling for covariates).

Individual sexual risk practices, detectable viral load, self-reported nonadherence and the computed HIV transmission risk outcome are reported in Table 1 (Table 1). Rates of sexual risk increased between baseline and follow-up. At baseline, nearly one in six participants (15.1%) reported at least one sexual risk in the last year, and at follow-up, over one-quarter (27.7%) reported any sexual risk. Viral load measures were available for *n* = 540 participants at baseline (55.2%) and *n* = 522 at follow-up (53.3%). The distribution of viral load values among participants with available data did not vary significantly by wave (Figure 1, Supplementary Digital Content 1). Of participants with available viral loads, 23% had detectable viral load at levels of high risk for sexual transmission (>1500 cop-ies/ml) at baseline and 18.8% at follow-up. Having no viral load results in medical records during the study period was strongly associated with self-reported defaulting and past-week nonadherence at both timepoints, controlling for socio-demographic covariates (Tables 1 and 2, Supplementary Digital Content 1). Adolescents included at both time points were more likely to report lower viremia at baseline compared with those who dropped out of the study (29.3 vs. 46.3%, *P* = 0.005), but no significant differences in sexual risk were documents (Table 3, Supplementary Digital Content 1).

**Table 3:** Predictors of secondary HIV transmission among antiretroviral therapy initiated South African adolescents living with HIV (*n* = 979).

	Model 1 All factors		Model 2 *P* < 0.10	
Baseline factors	OR (95% CIs)	*P*	OR (95% CIs)	*P*
Age binary 15+ years	2.05 (1.07–3.92)	0.030	2.51 (1.42–4.44)	0.002
Sex female	0.90 (0.53–1.54)	0.708		
Rural residence	1.01 (0.58–1.73)	0.991		
Poverty	1.29 (0.65–2.57)	0.469		
Double orphan	1.23 (0.67–2.24)	0.508		
School absenteeism ≥1 week	0.94 (0.52–1.68)	0.826		
Negative peer norms	1.02 (0.88–1.18)	0.813		
Internalized stigma	0.89 (0.46–1.71)	0.716		
Suicidality	1.35 (0.54–3.39)	0.526		
Substance/drug use	2.46 (1.26–4.81)	0.009	2.19 (1.19–4.02)	0.012
Household food insecurity	1.79 (1.05–3.06)	0.032	1.93 (1.18–3.14)	0.008
Positive caregiving	1.27 (0.77–2.11)	0.346		
Good caregiving monitoring	0.83 (0.47–1.48)	0.529		
Good caregiver communication	0.78 (0.45–1.35)	0.370		
Time on treatment 3+ years	1.19 (0.66–2.18)	0.582		
Knows HIV status	1.61 (0.78–3.34)	0.201		
Supported by treatment buddy	1.02 (0.59–1.76)	0.937		
Sexually infected	2.68 (1.44–4.99)	0.002	2.79 (1.66–4.68)	<0.001
Early sexual debut	2.47 (1.39–4.38)	0.002	2.40 (1.42–4.06)	0.001
Parenthood	5.22 (2.25–12.1)	<0.001	4.30 (2.16–8.57)	<0.001
Power-inequitable relationships	1.76 (1.02–3.04)	0.042	1.77 (1.08–2.92)	0.025

Rates of viremia were high but comparable at both timepoints: 30.4% of participants at baseline and 29.4% at follow-up reported either a viral load more than 1500 copies/ml or past-week ART nonadherence. Secondary transmission risk rates increased from 6.7% at baseline to 11.2% at follow-up, driven by increases in sexual risk. Although rates of transactional sex, unprotected sex and early pregnancy increased by several percentage points between two timepoints, the increase in sexual risk was driven by the near doubling of the rates of sex with and older partner and having multiple sexual partners in the last year. About 85.8% (*n* = 840) of all participants reported no HIV transmission risk at baseline and follow-up and were coded as *low risk* for the rest of the analyses.

### Characteristics of AYLHIV at high secondary transmission risk

Table 2 (Table 2) summarizes participant baseline characteristics for the full sample and two sub-groups: low and high secondary transmission risk. Participants were young adolescents at baseline with only 37% aged 15 years and older. Half the sample was female, with just over a quarter living in rural residences. Four out of five households reported limited access to basic necessities such as clothing, medicines and housing. One in five adolescents reported missing school, and one in six had lost both parents. Adolescents at high risk of secondary transmission were more likely to have been older, female, live in a poorer household, experience orphanhood and miss school, at baseline.

Over three-quarters of the adolescents reported internalized stigma, with 4% reporting suicidality and 8.6% reporting risky substance use. Around half of participants received positive caregiving and strong supervision, with only 26.8% reporting good communication with their caregivers. A quarter of participants reported hunger, which was nearly double among adolescents at high risk of HIV transmission.

Two-thirds of participating adolescents had been on treatment for 3 years or longer, yet only 67.6% knew their status. One in five was estimated to be sexually/recently infected and 71.7% said that they had a treatment buddy. Adolescents in the high-risk group were more likely to have recently initiated treatment, know their HIV status and be sexually infected. One in seven adolescents reported early sexual debut, with only 6.7% having a child; however, one in five reported being in power-inequitable relationships.

### Baseline factors associated with high secondary transmission risk

In multivariate regression analyses, older age, hunger, sexual HIV infection, power-inequitable relationships, early sexual debut, parenthood and substance use were associated with higher odds of secondary transmission risk. Compared with AYPLHIV at low risk at both time points, older adolescents (15–19 years old) and those who initiated sex before the age of 16 were more likely to report high risk of secondary transmission. Participants who reported ever being hungry were twice as likely to be in the high-risk group [odds ratio (OR) 1.93, 95% confidence interval (95% CI) 1.18–3.14, *P* = 0.008]. Sexually infected AYPLHIV were more than twice as likely to be in the high-risk group (OR 2.79, 95% CI 1.66–4.68, *P* < 0.001). Participants who reported substance use were also more likely to be in the high-risk group (OR 2.19, 95% CI 1.19–4.02, *P* = 0.012). Adolescents who had become parents were more than four times more likely to be at high risk of secondary transmission (OR 4.30, 95% CI 2.16–8.57, *P* < 0.001; Table 3).

Marginal effect modelling presented in Fig. 1 highlights the probability of reporting high secondary transmission risk, with all other variables kept at mean values. Without any of the factors, only 4.2% of the participants were likely to report being at high risk. AYPLHIV experiencing only one predictor were slightly more likely to be at high secondary transmission risk (7.2–15.6%). Parenthood was the largest single contributing factor to increases in the likelihood of being at high risk (+11.3%, from 4.3 to 15.6%). Among AYPLHIV who experienced all these factors, 88.9% of participants were likely to be in the high-risk group.

## Discussion

Maintaining an undetectable viral load while on ART protects against sexual transmission of HIV to others [43], also known as Undetectable = Untransmittable (U = U). However, we lack evidence on how to reach this goal for AYPLHIV. Encouraging AYPHLIV to maintain safe sexual practices and remain virally suppressed is crucial for their protection, as well as partners and children. In this cohort of AYPLHIV in South Africa, rates of secondary transmission risk nearly doubled over an 18-month period, as adolescents grew older, navigated HIV care and engaged in new relationships. Notably, at both timepoints, around one-third of AYPLHIV experienced viremia (using our composite measure), mirroring findings from a recent nationally representative survey [44]. Although rates of ART nonadherence and viremia remained high yet stable, rates of high-risk sexual activity increased from 15% at baseline to 28% at follow-up, again, echoing increases in age-related sexual risk documented in similar studies in South Africa and regionally [29]. Service providers should be supported to identify and address the one in six adolescents at high risk of secondary HIV transmission [44].

Our analyses found several factors significantly associated with secondary transmission risk: older age, early sexual debut, sexual HIV infection, hunger, difficult relationships, parenthood and substance use. Older adolescents were likelier to report high HIV transmission risk. Few studies report data from both sexually and vertically infected AYPLHIV. Our study documents how sexual infection was strongly associated with high transmission risk among adolescents, suggesting a continuum of risk starting with factors that expose younger adolescents to HIV, result in high-risk sexual relationships and infection, and extend to secondary transmission risk.

Hunger was significantly associated with higher secondary transmission risk, highlighting how food security can reduce sexual risk-taking and improve ART outcomes. Programming that improves access to food and nutritional supplements may have positive effects in reducing the risk of HIV exposure, and also the risk of secondary HIV transmission.

Our findings have implications for reducing secondary HIV transmission risk among AYPLHIV. First, in settings with limited access to timely viral load testing (<50% in this sample from South Africa), alternative models of identifying and support AYLHIV to reduce secondary HIV transmission are needed.

Second, screening for several key factors can support healthcare providers to identify AYPLHIV at risk of secondary transmission to partners and children. Our analyses show that combining three factors (hunger, parenthood and recent HIV infection) was highly sensitive; one in two participants experiencing these three factors would be at risk of secondary transmission. Healthcare providers could engage in simple conversations, and review clinical records, to determine both factors in routine care settings, and provide enhanced counselling and tailored interventions to reduce risk.

Third, it is vital to link sexually infected AYPLHIV to SRH services, especially adolescent girls and young women who find out about their HIV-positive status during pregnancy. For recently infected AYPLHIV, the immediate period following HIV diagnosis may be tumultuous, as they come to terms with their status [37] and navigate the sexual risk environment that may have caused their infection [45,46]. Although several successful models of integrating HIV and SRH services have been documented [47], evidence on the best models for adolescents, and specifically AYPLHIV, is needed. As healthcare systems have become overburdened in the context of the COVID-19 pandemic, integrating HIV and SRH services could help increase efficiency of patient-provider interactions [48].

Fourth, support for adolescent parents is important to address the syndemic of HIV and early parenthood. Small-scale programmes currently support adolescent girls and young women living with HIV to remain retained in care once they become mothers [49]. However, additional research is needed to identify scalable programming for this vulnerable group.

Fifth, although substance use was not common in our sample, it was very strongly associated with high risk of secondary transmission. Discussing risky substance use in nonjudgmental conversations during routine visits could be highly sensitive in identifying AYPLHIV most at risk for secondary transmission. However, given power differentials between adolescents and young people and healthcare providers, such screening may not work. School-based and parenting interventions known to reduce substance use in adolescents may help reduce secondary HIV transmission risk among AYPLHIV [50,51].

Sixth, AYPLHIV who were in power-inequitable relationships were more likely to be at risk of secondary transmission, highlighting these relationships’ roles in both primary and secondary prevention. With possible overlaps among HIV-positive status, age-disparate relationships, transactional sex and early parenthood, screening for recent HIV infection and parenthood may identify AYPLHIV at risk of secondary HIV transmission. Early relationships are often tentative and fluid; consequently, AYPLHIV have been largely omitted from interventions and research supporting sero-discordant adult couples to negotiate safe sex and support adherence to ART. Enhancing existing interventions that effectively support AYPLHIV to negotiate relationships is a necessary next step.

Finally, AYPLHIV are part of the continuum of our response to HIV, starting with early HIV testing for all and enrolment in treatment and care for those who are living with HIV. This study supplies additional evidence on a missing part of the transmission cycle, the partners of adolescents who are HIV-free but at risk of HIV infection.

Our study had several limitations. We only measured intimate partner violence – a factor previously reported to be associated with HIV transmission risk – at follow-up, and were unable to include it in analyses. Given high levels of missing viral load data in this context of an overburdened health system, viremia was calculated using a combination of viral load data and self-reported nonadherence. However, we conducted sensitivity analyses of various combinations of this measure; each identified the same factors associated with belonging to the high transmission-risk group. More rigorous and precise measures – such as timely viral load data and STI test results – would facilitate assessment of actual secondary HIV transmission risk for each individual. However, such data are not available in the study setting, nor in the routine care that these adolescent study participants can access. Further, although we applied rigorous analytic approaches, the study was conducted in only one country. However, the study setting and communities were selected because they reflect contexts where most AYPLHIV reside. However, our study is a large longitudinal study from a resource-constrained setting in South Africa, which has followed young adolescents from baseline into youth, allowing for this unique analysis.

Simple screening tools are needed to support healthcare providers, peers supporters and counsellors to identify at-risk AYPLHIV and to provide responsive HIV care, even where access to timely viral load data is infeasible. We identify several factors that can be easily screened to identify AYPLHIV most at risk of not realizing U = U. Although these items are not currently included in routine visits in public healthcare facilities, their inclusion is feasible and could help to provide differentiated services. Our findings suggest an urgent need to focus on early identification and intensified, tailored support for adolescents who have been recently sexually infected, who have become parents and who experience hunger, as well as those who reported substance use and power-inequitable relationships. These targeted efforts are key to getting closer to U = U among AYPLHIV and breaking the cycle of HIV transmission in this vulnerable age group.

## Acknowledgements

E.T. and L.C. designed the overall study, including data collection tools. E.T. conceptualized and carried out analyses with support from S.Z. and R.H. and wrote the manuscript draft. C.L., W.S. reviewed the literature and contributed to results interpretation. L.G. advised on focus and framing of research. E.T., R.H. and L.C. designed and led the medical records data collection, cleaning and analyses. All authors have reviewed and approved this manuscript. We are grateful to all study participants, their families and healthcare providers, who opened their hearts, minds and personal/professional spaces to the study team, the Teen Advisory Group and the research study team. We are additionally grateful to our teams at the Universities of Cape Town and Oxford, A/Prof. Rebecca Hodes, Prof. Mark Orkin for his long-standing advice on analyses, Ms Rachel Pearson, Dr. Natella Rakhmanina, Prof. Don Operario, A/Prof. Catherine Mathews and A/Prof. Abigail Harrison.

### Conflicts of interest

The project has been funded by the International AIDS Society through the CIPHER grant (155-Hod; 2018/625-TOS). The views expressed in written materials or publications do not necessarily reflect the official policies of the International AIDS society; the Claude Leon Foundation [F08 559/C]; Evidence for HIV Prevention in Southern Africa (EHPSA), a UK aid programme managed by Mott MacDonald; Janssen Pharmaceutica N.V., part of the Janssen Pharmaceutical Companies of Johnson & Johnson; Nuffield Foundation, but the views expressed are those of the authors and not necessarily the Foundation (Visit www.nuffieldfoundation.org); the Oak Foundation grants [R46194/AA001] and [OFIL-20–057]; the Regional Inter-Agency Task Team for Children Affected by AIDS - Eastern and Southern Africa (RIATT-ESA); the John Fell Funds [161/033] and [103/757]; the Leverhulme Trust (PLP-2014–095); University of Oxford's ESRC Impact Acceleration Account (IAA) [1602-KEA-189] and [K1311-KEA-004]; UNICEF Eastern and Southern Africa Office (UNICEF-ESARO); UKRI GCRF Accelerating Achievement for Africa's Adolescents (Accelerate) Hub (Grant Ref: ES/S008101/1). E.T. was supported by the Fogarty International Center, National Institute on Mental Health, National Institutes of Health under Award Number K43TW011434. The content is solely the responsibility of the authors and does not represent the official views of the National Institutes of Health. Funding was also provided for the research team by the European Research Council (ERC) under the European Union's Seventh Framework Programme [FP7/2007-2013]/ ERC grant agreement no. 313421, the European Union's Horizon 2020 research and innovation programme/ERC grant agreement no. 737476). E.T. was supported by the Oxford University Clarendon-Green Templeton College Scholarship in 2012-2016.

The views expressed in written materials or publications do not necessarily reflect the official policies of the International AIDS society; the Claude Leon Foundation [F08 559/C]; Evidence for HIV Prevention in Southern Africa (EHPSA), a UK aid programme managed by Mott MacDonald; Janssen Pharmaceutica N.V., part of the Janssen Pharmaceutical Companies of Johnson & Johnson; Nuffield Foundation, but the views expressed are those of the authors and not necessarily the Foundation. Visit www.nuffieldfoundation.org; the Oak Foundation grants [R46194/AA001] and [OFIL-20–057]; the Regional Inter-Agency Task Team for Children Affected by AIDS - Eastern and Southern Africa (RIATT-ESA); the John Fell Funds [161/033] and [103/757]; the Leverhulme Trust (PLP-2014–095); University of Oxford's ESRC Impact Acceleration Account (IAA) [1602-KEA-189] and [K1311-KEA-004]; UNICEF Eastern and Southern Africa Office (UNICEF-ESARO); UKRI GCRF Accelerating Achievement for Africa's Adolescents (Accelerate) Hub (Grant Ref: ES/S008101/1). Research reported in this publication was supported by the Fogarty International Center, National Institute on Mental Health, National Institutes of Health under Award Number K43TW011434. The content is solely the responsibility of the authors and does not represent the official views of the National Institutes of Health.

## Supplementary Material

Supplemental Digital Content
