# Supplementary material for: Predictors of secondary HIV transmission risk in a cohort of adolescents living with HIV in South Africa
Source: AIDS. 2021 Aug 9;36(2):267–76. doi: 10.1097/QAD.0000000000003044 (PMC8702447; doi:10.1097/QAD.0000000000003044)
Supplement: Supplemental Digital Content [file aids-36-267-s001.docx]

# Predictors of secondary HIV transmission risk in a cohort of adolescents living with HIV in South Africa

Supplementary Material

**Supplementary figure 1: Distribution of viral load values among participants with available data (n=540 in Wave 1 and n=522 in Wave 2)**

**Supplementary table 1: Association between missing viral load and self-reported defaulting: Full sample**

|  | **Baseline (N=1 046)** | | **Follow-up (N=1 030)** | |
| --- | --- | --- | --- | --- |
| **Factors** | **OR (95% CIs)** | **p-value** | **OR (95% CIs)** | **p-value** |
| Defaulting | **3.80 (2.0-7.23)** | **<0.001** | **1.82 (1.24-2.68)** | **0.002** |
| Age (15+ years) | **1.45 (1.12-1.88)** | **0.005** | **1.86 (1.44-2.41)** | **<0.001** |
| Gender (female) | 1.15 (0.90-1.49) | 0.269 | 1.28 (0.99-1.66) | 0.058 |
| Rural residence | 0.95 (0.71-1.26) | 0.706 | 0.95 (0.71-1.28) | 0.741 |
| Informal housing | 0.83 (0.60-1.14) | 0.246 | 0.74 (0.51-1.06) | 0.100 |
| Poverty | 1.09 (0.83-1.42) | 0.536 | 0.86 (0.64-1.17) | 0.334 |

**Supplementary table 2: Association between missing viral load record and past-week non-adherence: Full sample**

|  | **Baseline (N=1 046)** | | **Follow-up (N=1 030)** | |
| --- | --- | --- | --- | --- |
| **Factors** | **OR (95% CIs)** | **p-value** | **OR (95% CIs)** | **p-value** |
| Past-week non-adherence | **1.62 (1.25-2.11)** | **0.000** | **1.63 (1.25-2.13)** | **0.000** |
| Age (15+ years) | **1.52 (1.18-1.97)** | **0.001** | **1.90 (1.47-2.46)** | **0.000** |
| Gender (female) | 1.19 (0.92-1.53) | 0.178 | **1.34 (1.04-1.73)** | **0.026** |
| Rural residence | 0.89 (0.67-1.19) | 0.435 | 0.97 (0.72-1.30) | 0.834 |
| Informal housing | 0.85 (0.62-1.18) | 0.331 | 0.74 (0.52-1.07) | 0.107 |
| Poverty | 1.09 (0.83-1.42) | 0.544 | 0.88 (0.65-1.20) | 0.419 |

**Supplementary table 3: Baseline characteristics of the full sample (n=1046)**

| **Baseline Characteristics** | **Full sample**  **(N=1046)** | **Baseline participants included at follow up**  **(n-979)** | **Baseline participants excluded at follow-up (N=67)** |  |
| --- | --- | --- | --- | --- |
|  | **N (%)** | **N (%)** | **N (%)** | **p-value** |
| **Sexual risk factors** |  |  |  |  |
| Transactional sex | 68 (6.5%) | 65 (6.6%) | 3 (4.5%) | 0.487 |
| Sex with older partner | 26 (2.5%) | 26 (2.7%) | 0 (0%) | 0.177 |
| Unprotected sex | 65 (6.2%) | 62 (6.3%) | 3 (4.5%) | 0.543 |
| Multiple sexual partners | 91 (8.7%) | 88 (9.0%) | 3 (4.5%) | 0.205 |
| Pregnancy | 75 (7.2%) | 69 (7.1%) | 6 (8.9%) | 0.558 |
| *Any sexual risk (combined)* | *158 (15.1%)* | *149 (15.2%)* | *9 (13.4%)* | *0.693* |
| **Treatment-related outcomes** |  |  |  |  |
| Detectable viral load (>200 copies/mL)^¥^ | 167 (29.0%) | 150 (27.8%) | 17 (46.0%) | **0.018** |
| Detectable viral load (>1500 copies/mL)^¥^ | 124 (23.0%) | 111 (20.6%) | 13 (35.1%) | **0.037** |
| Past-week non-adherence | 365 (34.9%) | 335 (34.2%) | 30 (44.8%) | 0.079 |
| *Viremia** | *318 (30.4%)* | *287 (29.3%)* | *31 (46.3%)* | ***0.005*** |
| **Secondary HIV transmission risk** | **70 (6.7%)** | **63 (6.4%)** | **7 (10.5%)** | **0.204** |

*****Combination of past-week non-adherence for those without VL record and detectable viral load (>1,000 copies/mL) for those with VL record.

**^¥^** Viral load data were available for 577 at baseline and 522 at follow-up. Frequencies of detectable viral load computed on available data.
† Secondary HIV transmission risk computed as participants reporting both sexual risk and viremia at each time point.

**Supplementary table 4: Multicollinearity checks using variance inflation factors (VIFs) for factors evaluated in multivariable multiple regression (>=1500 copies/mL)**

| **Variable** | **VIF** | **Tolerance (1/VIF)** |
| --- | --- | --- |
| Age (binary) | 2.82 | 0.355 |
| Sex (female) | 2.32 | 0.431 |
| Rural residence | 1.40 | 0.714 |
| Poverty | 4.24 | 0.236 |
| Double orphan | 1.26 | 0.793 |
| School absenteeism (>= 1 week) | 1.50 | 0.664 |
| Negative peer norms | 2.35 | 0.425 |
| Internalized stigma | 3.54 | 0.283 |
| Suicidality | 4.15 | 0.241 |
| Substance/drug use | 1.13 | 0.883 |
| Household food insecurity | 1.37 | 0.732 |
| Positive caregiving | 2.20 | 0.454 |
| Good caregiver monitoring | 1.98 | 0.505 |
| Good adolescent-caregiver communication | 1.43 | 0.698 |
| Time on treatment (3+ years) | 1.38 | 0.723 |
| Knows HIV status | 3.56 | 0.281 |
| Treatment buddy support | 3.89 | 0.257 |
| Sexually infected | 2.17 | 0.461 |
| Early sexual debut | 1.60 | 0.625 |
| Parenthood | 1.66 | 0.601 |
| Power-inequitable relationships | 1.66 | 0.602 |
